# The Prevalence of Impulse Control Disorders and Behavioral Addictions in Eating Disorders: A Systematic Review and Meta-Analysis

**DOI:** 10.3389/fpsyt.2021.724034

**Published:** 2022-01-06

**Authors:** Daniel J. Devoe, Alida Anderson, Anees Bahji, Manya Singh, Scott B. Patten, Andrea Soumbasis, Ana Ramirez Pineda, Jordyn Flanagan, Candice Richardson, Tom Lange, Gina Dimitropoulos, Georgios Paslakis

**Affiliations:** ^1^Mathison Centre for Mental Health Research and Education, Department of Psychiatry, University of Calgary, Calgary, AB, Canada; ^2^Faculty of Social Work, University of Calgary, Calgary, AB, Canada; ^3^Ruhr-University Bochum, University Clinic for Psychosomatic Medicine and Psychotherapy, Lübbecke, Germany

**Keywords:** eating disorders, behavioral addictions, impulse control disorders (ICD), comorbidity, systematic review

## Abstract

**Aim:** Individuals with eating disorders (EDs) may present with impulse control disorders (ICDs) and behavioral addictions (BAs), which may result in additional suffering and treatment resistance. However, the prevalence of ICDs and BAs in EDs has not been systematically examined. Therefore, this systematic review and meta-analysis aimed to assess the prevalence of ICDs and BAs in ED samples.

**Methods:** A comprehensive electronic database search of the peer-reviewed literature was conducted in the following online databases: MEDLINE, PsycINFO, Embase, and CINAHL from their inception to May 2021. We restricted review eligibility to research studies reporting prevalence for ICDs or BAs in individuals with diagnosed EDs. The outcome for this review was the prevalence of ICDs or BAs in individuals with EDs. A series of random-effects meta-analyses were performed on eligible studies to estimate the pooled proportions and 95% confidence intervals (CIs).

**Results:** Thirty-five studies met the inclusion criteria, including a total of 9,646 individuals identified as having an ED, 18 of these studies specifically examined ICDs/BAs in AN, BN, and BED. Random-effects pooled estimates demonstrated that the comorbid prevalence of any ICD was 22%. The prevalence of comorbid pathological/compulsive buying was highest (19%), followed by kleptomania (18%), pathological internet use (12%), intermittent explosive disorder (4%), trichotillomania (3%), and gambling disorder (2%). In addition, the prevalence of stealing/shoplifting behaviors was 30% in those with EDs.

**Conclusion:** This is the first meta-analysis on the comorbid prevalence of EDs and ICDs/BAs. We found a moderate prevalence for these comorbid conditions, with approximately one out of five individuals with an ED also displaying a comorbid ICD/BA. Although causal inferences cannot be drawn, the numbers strongly suggest that clinical screening/monitoring of ICDs/BAs should be part of the clinical routine in cohorts with EDs. ED settings need either the capacity to manage these disorders or adequate access to relevant services. Further investigations are needed to reveal common underlying pathomechanisms.

**Systematic Review Registration:** https://www.crd.york.ac.uk/prospero/, identifier: CRD42020202044.

## Introduction

Eating disorders (EDs) are severe and life-threatening psychiatric conditions with significant medical (e.g., cardiovascular, renal, and gastrointestinal) and psychological consequences that include an increased risk of suicide (1–4). EDs involve using restrictive or purging behaviors to regulate one's eating or to control weight and to manage negative attitudes and body image distortions (5). The most common EDs are anorexia nervosa (AN), bulimia nervosa (BN), and binge eating disorder (BED) (6). Still, they can also encompass other specified feeding and eating disorders, such as avoidant restrictive food intake disorder, pica, and rumination disorder. The lifetime prevalence of EDs in the general population has been reported to be as high as 5% (7–9), with a lower lifetime prevalence reported for AN (0.6%), 1.0% for BN, and a higher lifetime prevalence of 2.8% for BED (10). Furthermore, EDs incur high healthcare costs (2) and are associated with greater healthcare utilization, high rates of hospitalization, and increased emergency department visits (11).

Impulse control disorders (ICDs), characterized by repetitive or compulsive behaviors with negative consequences, stem from the inability to resist impulses or urges before engaging in the behavior or deriving a sense of gratification from engaging in the behavior (12). ICD subtypes are numerous and include gambling disorder, kleptomania, pyromania, compulsive sexual disorder, and intermittent explosive disorder (13). Closely related to ICDs are a subset of conditions known as Behavioral Addictions (BAs), described as sharing features with ICDs and substance use disorders (14). BAs refer to an addiction of a behavioral component rather than the ingestion of a substance with psychoactive properties (15) and currently include Gambling Disorder and Internet Gaming Disorder in the DSM-5. However, other known presentations of BAs are not represented in the DSM-5 yet are extensively explored in the literature, including technological addiction, sex addiction, compulsive buying, and work addiction (16). Similar to EDs, prevalence estimates of ICDs and BAs across the lifespan are high, ranging from 0.6 to 11.1% (17) and 1.2 to 8% (18, 19) across diagnostic categories for ICDs and BAs, respectively.

Research has focused on EDs and comorbid conditions in the past two decades to examine how concomitant disorders have influenced the illness course and pathways to and participation in treatment. Current estimates suggest that up to 70% of individuals with EDs have psychiatric comorbidities (20), including mood and anxiety disorders, personality disorders, and substance use disorders (5, 21). The presence of psychiatric comorbidities in EDs are costly, which may be associated with longer lengths of stay in treatment programs, higher dropout rates from programs, and poorer treatment outcomes, and tend to be mostly higher in individuals with severe and enduring EDs (20, 22). Hence, also the comorbidity between EDs and ICDs has been examined.

EDs are postulated to share similar personality profiles and neurobiological mechanisms contributing to the etiology and psychopathology of these disorders, such as emotional dysregulation, personality traits like high levels of novelty seeking and high levels of sensitivity to punishment and reward, and compulsivity. Research demonstrates that individuals with EDs present with higher rates of impulsivity and impulsive behaviors, including self-injury, substance use, stealing, and unsafe sexual practices (3, 12, 23–30). Similarly, EDs (particularly those with Bulimia Nervosa) and other BAs such as compulsive buying and gambling disorder share a propensity for impulsivity; for example, adults with compulsive buying have been shown to exhibit higher symptomatology of BN and BED compared to healthy controls (31), sharing low levels of inhibitory and activation control (32). Furthermore, individuals who present with comorbidities of EDs and BAs show a higher burden of general psychopathology and higher novelty-seeking (33). Other studies demonstrate changes in reward centers of the brain, e.g., in ventral striatal activity (34), which are crucial elements to the neurobiological profiles of EDs, ICDs, and BAs (35). There is also substantial empirical evidence supporting frameworks for a dimensional categorization of both behavioral addictions as well as eating disorders on the impulsive-compulsive spectrum (36). Finally, examining the comorbidity of eating disorders and behavioral addictions is important to design and implement novel therapeutic strategies in addition to established treatments (e.g., CBT). Novel strategies may address the above mentioned common underlying vulnerabilities (e.g., impulsivity or emotional dysregulation).

Despite a growing body of literature that demonstrates similarities in the underlying mechanisms and etiology of these disorders, no systematic review or meta-analysis has examined the prevalence, associations, and quality of the literature of studies conducted on ICDs or BAs in those with EDs. Systematic reviews and meta-analyses can explore heterogeneity and pool estimates when differences are due to sampling variability. Therefore, this systematic review and meta-analysis aimed to (1) assess the prevalence of ICDs or BAs in those diagnosed with EDs; (2) assess the prevalence of ICDs or BAs by ED subtype (e.g., AN, BN, and BED), and (3) assess the quality of peer-reviewed literature to date.

## Methods

### Protocol

This systematic review and meta-analysis was registered with the PROSPERO database of systematic reviews (CRD42020202044). This review followed both the meta-analysis of observational studies in epidemiology (MOOSE) recommendations (37) and the preferred reporting for systematic reviews and meta-analyses (PRISMA) guidelines (38, 39).

### Search Strategy

A comprehensive electronic database search of the peer-reviewed literature was conducted in the following online databases: MEDLINE, PsycINFO, Embase, and CINAHL from their inception to May 2021. The key words included two concepts: (1) various EDs [i.e., anorexia nervosa (AN), bulimia nervosa (BN), binge eating disorder (BED), or eating disorders not otherwise specified (EDNOS)] and (2) ICDs/BA (i.e., impulse control disorder total, pathological/compulsive buying, kleptomania, trichotillomania, intermittent explosive disorder, pyromania, pathological/compulsive gambling, technology addictions, and stealing/shoplifting). The electronic database searches and exhaustive list of keywords are provided in the Supplementary Material. Two blinded reviewers (A.A. and A.P.) independently performed title, abstract and full-text article screening using the online Covidence systematic review software (40). The reference lists of included articles were hand-searched for other relevant studies that might meet the inclusion criteria and were not found through online database searching.

### Selection Criteria

Peer-reviewed studies were selected by two reviewers (A.A. and A.P.) for inclusion in this review if they met the following criteria: (1) research including participants with any EDs, anorexia nervosa (AN), bulimia nervosa (BN), binge eating disorder (BED), or eating disorders not otherwise specified (EDNOS) or reported total EDs, based on any DSM/ICD criteria; (2) reported on ICDs or BAs either clinician reported or self-reported (i.e., impulse control disorder total, pathological/compulsive buying, kleptomania, trichotillomania, intermittent explosive disorder, pyromania, pathological/compulsive gambling, technology addictions, and stealing/shoplifting); (3) reported the prevalence of ICDs or BAs in EDs, (4) contained cross-sectional data for the meta-analysis. In addition, this review excluded studies that: (1) looked at the relationship between EDs and substance abuse disorders; (2) had ineligible study designs such as case reports, review articles, opinion pieces, and editorials; and (3) had insufficient data for the meta-analysis. Disagreements were first discussed in a consensus meeting, and DD made the final decision for inclusion or exclusion.

### Data Extraction

Data abstraction was completed in duplicate (AA and AS), including the following study characteristics: author, year of publication, country, the control group, total sample size, types of ICDs or BAs, types of EDs, total ED sample size, age (mean ± SD), number of females/percent female, cohort or comparison group description, and outcomes reported. For the meta-analysis, the following data were extracted: (1) author, (2) year of publication, (3) ICDs or BAs type, (4) ED type, (5) numerator representing ICDs or BAs, and (6) the denominator representing EDs or ED subtype.

### Risk-of-Bias Assessment

Studies included in this systematic review and meta-analysis were evaluated for quality using a modified Downs and Black instrument (41) by one author (TL). The modified Downs and Black checklist for cross-sectional studies utilizes 14-items to evaluate the risk of bias and provides a total score out of 15 points for each study. Thus, higher scores indicate greater quality. In addition, this quality assessment checklist contains yes/no questions, which are applied to a series of questions regarding the quality of each study.

### Data Synthesis and Analysis

Due to the expected heterogeneity between studies, the estimates were initially stratified by ED subgroup, and a series of DerSimonian and Laird (42) random-effects meta-analyses were performed on eligible studies to estimate pooled proportions and 95% CIs for ICDs and BAs for all EDs within those strata. All meta-analyses in this review utilized the Freeman-Tukey double arcsine transformation, which computes the weighted pooled estimate using a variance-stabilizing transformation and then performs a back-transformation on the pooled estimate. This approach is favorable in situations where there is zero count data, as it prevents zero count studies from being dropped from the analyses creating a bias in prevalence estimates. The exact confidence interval method was used as this is considered more conservative. Statistical heterogeneity was calculated utilizing the *I*^2^ statistic for subgroup meta-analysis that included four or more studies. All analyses were performed in STATA v.17 (43). To avoid double-counting in the meta-analysis, the data from the largest sample (i.e., denominator) was utilized in the case of two or more studies reporting in the same sample. Forest plots were created showing the proportion of those with either ICDs or BAs in individuals with EDs. Subgroup meta-analyses were used to distinguish the prevalence of ICDs or BAs in EDs types.

## Results

### Search Yield

**Figure 1:**
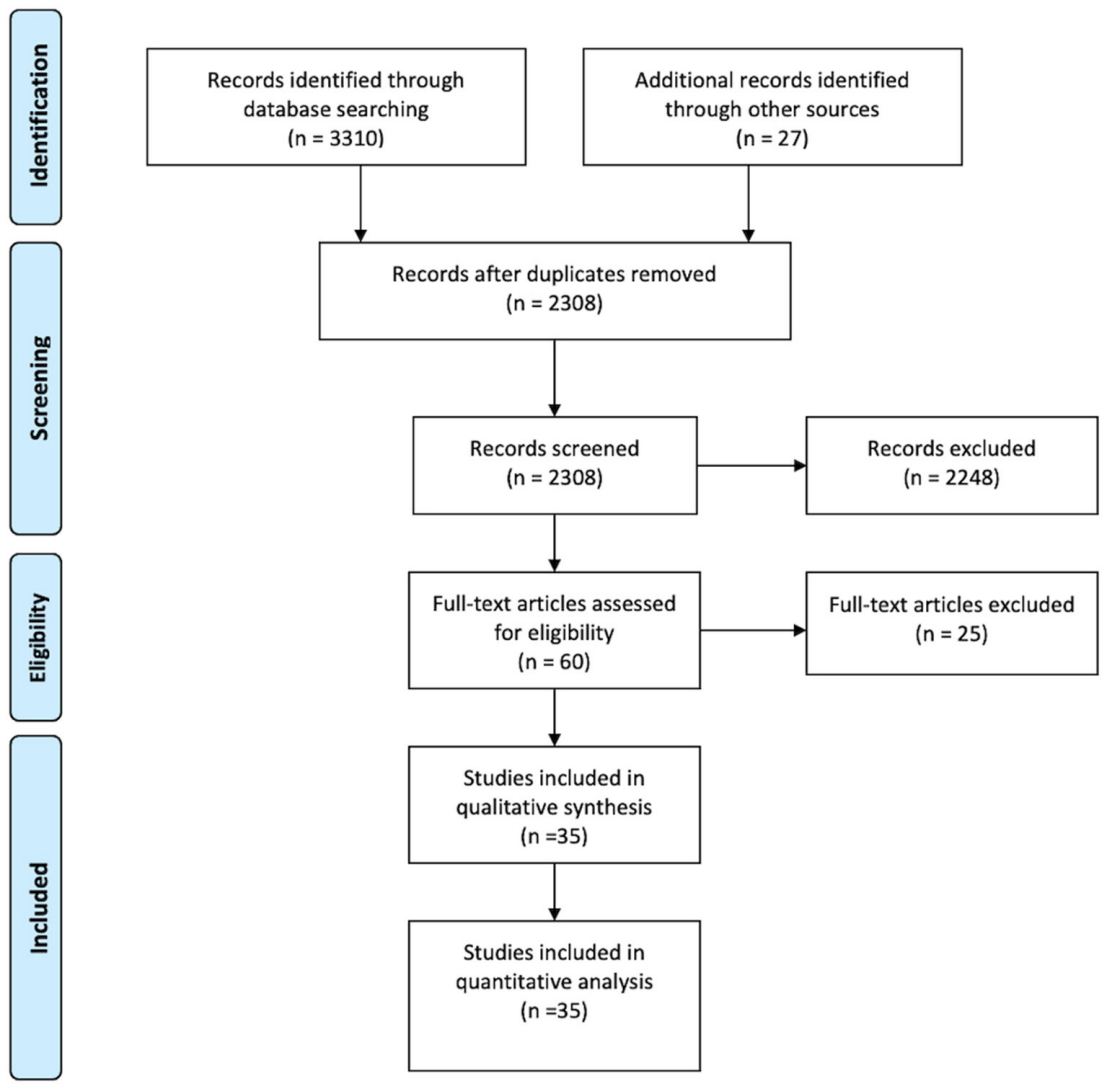
Screening flow diagram.

Electronic database searches identified 3,310 records; after duplicate references were removed, a total of 2,308 abstracts and titles were screened. The level of agreement between the two reviewers for screening titles and abstracts was moderate (κ = 0.60). After resolution of inconsistencies, a total of 60 studies were retrieved and reviewed in full text. Overall, 35 studies met the inclusion criteria and were included in the meta-analysis, see Figure 1.

### Study Characteristics

All studies included in this review are described in detail in Tables 1, 2. Studies were published between 1980 and 2019. Most studies were conducted in North America (*n* = 14), followed by Europe (*n* = 13) and Asia (*n* = 8). Twenty-two studies recruited ED individuals from a hospital setting or a specific eating disorder program, nine were recruited from outpatient clinics, and four were recruited from research study cohorts.

**Table 1:** Study and participant characteristics of included studies (*N* = 35).

**References**	**Country**	**Control**	**Total sample size**		**Type(s) of eating disorders**	**Eating disorder patients**		
				**Type(s) of behavioral addictions**		***N***	**Age M ± SD**	**Female** ***N*** **(%)**
Blinder et al. (44)	USA	n/a	2,436	Trichotillomania	520 AN-R 436 AN-P 870 BN-P 12 BN-NP 598 EDNOS	2,436	23.4 ± 8.6	2,436 (100%)
Bulik et al. (45)	USA	n/a	432	Shoplifting or stealing gambling Overspending Fire setting	AN-R AN-P AN-B ANBN	432	30.4 ± 11.3	410 (94.9%)
Casper et al. (46)	USA	n/a	75	Pathological gambling	AN	75	Early adolescent onset: 16.2 ± 3.3 Late adolescent onset: 19.7 ± 3.3 Adult onset: 25.2 ± 3.7	75 (100%)
Christenson et al. (47)	USA	Healthy controls (*n* = 40)	105	Trichotillomania Self-injurious behavior Compulsive stealing	65 BN	65	27 ± 6.28	65 (100%)
Claes et al. (32)	Belgium	n/a	60	Compulsive buying Compulsive internet use	23 AN-R 4 AN-BP 16 BN 17 EDNOS	60	27.82 ± 9.76	60 (100%)
Corstorphine et al. (48)	UK	n/a	102	Impulsive self-harm Compulsive stealing Compulsive spending	23 AN-R 19 AN-BP 40 BN 20 EDNOS	102	29.3 ± 8.98	101 (99.0%)
Crisp et al. (49)	UK	n/a	102	Stealing	AN	102	Shoplifters: 27.4 ± 7.94 Non-shoplifters: 20.1 ± 92	102 (100%)
de la Serna de Pedro et al. (50)	Spain	n/a	45	Kleptomania	BN	45	22.22 (median) (Range: 17–38)	40 (88.9%)
Eddy et al. (51)	USA	n/a	246	Kleptomania	24 AN-R “pure” 27 AN-R “not pure” 85 AN-BP 110 BN	246	ANR “pure”: 20.8 ANR “not pure”: 23.8 ANBP: 22.7 (SD not reported)	246 (100%)
Faber et al. (31)	USA	Obese non-binge eaters (*n* = 113)	197	Compulsive buying	BED	84	39.9 (SD not reported)	84 (100%)
Fernández-Aranda et al. (52)	Spain	Pathological gamblers (*n* = 42)	269	Impulse control disorders (ICD): Kleptomania Pyromania Pathological gambling Trichotillomania Compulsive buying	BN	227	BN-ICD: 25.7 ± 6.9 BN + ICD: 26.7 ± 6.7	227 (100%)
Fernández-Aranda et al. (12)	Spain	n/a	709	Impulse control disorders (ICD): Compulsive buying Kleptomania Trichotillomania Intermittent explosive disorder Compulsive gambling Pyromania	59 AN-R 29 AN-B 33 AN-P 252 BN-P 22 BN-NP 251 ANBN 63 EDNOS	709	Not reported Range: 13–65	709 (100%)
Fernández-Aranda et al. (36)	Spain	Gambling disorder (*n* = 184) Healthy controls (*n* = 151)	511	Pathological buying (PB)	AN BN BED OSFED	176	31.71 ± 12.84	157 (89.2%)
Gerlinghoff and Backmund (53)	Germany	n/a	63	Kleptomania, stealing	23 AN 4 BN 36 ANBN	63	20.4 (SD not reported)	Not reported
Goldner et al. (54)	Canada	Psychiatric controls (PCG *n* = 46) Undergraduate control group (UCG *n* = 82)	176	Shoplifting	23 AN 18 BN 7 EDNOS	48	27.1 ± 8.6	48 (100%)
Herzog et al. (55)	USA	n/a	229	Kleptomania	41 AN 98 BN 90 ANBN	229	AN: 22.8 ± 7.4 BN: 24.8 ± 6.1 ANBN: 26.1 ± 6.6	229 (100%)
Hudson et al. (56)	USA	First degree relatives of 17 probands with schizophrenia (*n* = 41) First degree relatives of 15 probands with bipolar disorder (*n* = 50)	181	Kleptomania Intermittent explosive disorder	16 AN 25 ANBN 49 BN	90	AN: 25.0 ± 7.0 (15 Females only, one male aged 24) ANBN = 25.8 ± 7.3 (24 females only, one man aged 28) BN: 28.4 ± 8.0 (46 females only, 3 males aged 26,41,56)	85 (94.4%)
Jiménez-Murica et al. (57)	Spain	n/a	1,681	Pathological gambling	354 AN 783 BN 105 BED 439 EDNOS	1,681	ED: 26.23 ± 7.5 ED + PG: 27.60 ± 7.3	1,576 (94%)
Jiménez-Murica et al. (33)	Spain	Non-psychiatric controls (*n* = 50) Compulsive buying (CB; *n* = 36) Gambling disorder (GD; *n* = 53)	238	Compulsive buying Gambling disorder	BN	99	BN-CB: 28.1 ± 8.2 BN + CB: 26.9 ± 8.1	99 (100%)
Lacey and Read (58)	UK	n/a	10	Stealing	BN	10	26.2 (SD not reported)	10 (100%)
Matsunaga et al. (27)	Japan	n/a	64	Shoplifting	33 BN 31 AN-B	64	BN + MI: 25.4 ± 4.2 BN – MI: 24.1 ± 3.0	64 (100%)
Miyawaki et al. (59)	Japan	n/a	284	Shoplifting	99 AN-R 72 AN-BP 113 BN	284	24.6 ± 7.0	284 (100%)
Nagata et al. (60)	Japan	Female student controls (*n* = 66)	302	Repeated shoplifting	60 AN-R 62 AN-BP 114 BN-P	236	AN-R: 22.3 ± 4.0 AN-BP:25.0 ± 5.1 BN-P: 22.6 ± 4.2	236 (100%)
Nagata et al. (61)	Japan	n/a	185	Shoplifting	62 AN-R 48 AN-BP 75 BN	185	ED + DUD: 25.1 ± 5.7 ED-DUD: 24.2 ± 5.5	185 (100%)
Nagata et al. (62)	Japan	Patients with methamphetamine use disorder (*n* = 12)	31	Shoplifting	2 AN-R 8 AN-BP 9 BN	19	ED + DUD: 24.8 ± 5.4	19 (100%)
Nozoe et al. (63)	Japan	n/a	55	Stealing	AN	55	17.5 (SD not reported) (Range: 12–27)	50 (90.9%)
Pryor et al. (64)	USA	n/a	171	Stealing	100 AN-R 71 AN-BP	171	22.66 ± 8.33	171 (100%)
Rowston and Lacey (65)	UK	n/a	312	Stealing	BN	312	Stealers: 25.2 Non-stealers: 24.9 (SD not reported)	312 (100%)
Takei et al. (66)	Japan	n/a	16	Kleptomania	AN	16	Not reported	15 (93.8%)
Tanaka et al. (67)	Japan	n/a	61	Shoplifting	27 AN-R 34 AN-BP	61	22.7 ± 6.0	61 (100%)
Vandereycken and Houdenhove (29)	Belgium	n/a	155	Stealing	51 AN-R 62 AN-Mixed 39 BN 3 EDNOS	155	AN-R: 23.5 ± 7.3 AN-mixed: 22.5 ± 5.7 BN:23.0 ± 3.8 EDNOS not reported	155 (100%)
Weiss and Ebert (68)	USA	Normal-weight female controls (*n* = 15)	30	Stealing Impulsive buying	BN	15	26.0 ± 4.3	15 (100%)
Wiederman and Pryor (30)	USA	n/a	217	Stealing	BN-P	217	25.05 ± 4.92	217 (100%)
Yip et al. (69)	USA	n/a	94	Pathological Gambling	BED	94	43.93 ± 8.94	66 (70.2%)
Zucker et al. (70)	USA	n/a	1,453	Trichotillomania	389 AN-R 215 AN-P 138 AN-BP 258 BN-P 22 BN-NP 65 EDNOS 366 ANBN	1,453	27.1 ± 8.5	Not reported

*AN, Anorexia nervosa; AN-BP, Anorexia nervosa- binge/purge subtype; AN-B, Anorexia nervosa binge subtype; AN-P, Anorexia nervosa purge subtype; AN-R, Anorexia nervosa- restricting subtype; BN, bulimia nervosa; BN-P, Bulimia nervosa with purging; BN-NP, Bulimia nervosa with binging (no purging); ANBN, lifetime diagnosis of AN and BN; EDNOS, eating disorder not otherwise specified; BED, Binge eating disorder; CB, compulsive buying; ICD, Impulse control disorder; ED, Eating disorder; TAU, treatment as usual; CBT, cognitive behavioral therapy; CBT-AN, cognitive behavioral therapy for AN; BN-P – MI, Bulimia Nervosa with purging with no multi-impulsivity; BN-P + MI, Bulimia Nervosa with purging with multi-impulsivity; ED + DUD, Eating disorder with comorbid drug use disorder; ED-DUD, Eating disorder without comorbid drug use disorder*.

**Table 2:** Details of eating disorders and behavioral addictions results of included studies (*N* = 35).

**References**	**Cohort or comparison groups**	**Outcomes**
Blinder et al. (44)	•Female inpatients treated for AN, BN, or EDNOS between January 1, 1995, and December 31, 2000 (*n* = 2,436)	•11/2,436 (0.5%) of total ED patients had comorbid trichotillomania •2/520 (0.4%) AN-R patients had comorbid trichotillomania •3/436 (0.7%) AN-B patients had comorbid trichotillomania •3/882 (0.3%) BN patients had comorbid trichotillomania •3/598(0.5%) EDNOS patients had comorbid trichotillomania
Bulik et al. (45)	•First 432 consecutive people enrolled in the NIH funded Genetics of Anorexia Nervosa Collaborative Study (*n* = 432)	•42/412 (10.2%) of all assessed AN patients reported shoplifting or stealing •29/343 (8.5%) of AN patients with no suicide attempts reported shoplifting or stealing •13/69 (18.8%) of AN patients with at least one suicide attempt reported shoplifting or stealing •1/412 (0.24%) of all assessed AN patients reported gambling •1/343 (0.3%) of AN patients with no suicide attempts reported gambling •0/69 (0%) of AN patients with at least one suicide attempt reported gambling •2/412 (0.5%) of all assessed AN patients reported fire setting •1/343 (0.3%) of AN patients with no suicide attempts reported fire setting •1/69 (1.5%) of AN patients with at least one suicide attempt reported fire setting •46/412 (11.2%) of all assessed AN patients reported over spending •32/343 (9.4%) of AN patients with no suicide attempts reported over spending •14/69 (20.9%) of AN patients with at least one suicide attempt reported over spending
Casper et al. (46)	•Females diagnosed with AN (*n* = 75)	•No AN patient had lifetime diagnosis of pathological gambling at the 8 year follow-up
Christenson et al. (47)	•Adult women diagnosed with BN (*n* = 65) •Adult women controls (*n* = 40)	•There was no difference in prevalence of trichotillomania among adult women with BN and controls •There was a trend toward higher prevalence of compulsive stealing in subjects with BN
Claes et al. (32)	•Female outpatients diagnosed with AN, BN, or EDNOS (*n* = 60)	•10% prevalence of compulsive buying and compulsive internet use •Compulsive buying was related to restrictive eating behaviors and bulimic symptoms; whereas, compulsive internet use was only related to restrictive eating behaviors •There was a strong positive association between compulsive buying and compulsive internet use •Compulsive buying and internet use showed positive correlations with emotional liability, excitement seeking and lack of effortful control
Corstorphine et al. (48)	•Individuals who met DSM-IV criteria for an eating disorder (*n* = 102)	•9/102 (8.8%) of ED patients reported compulsive spending •8/102 (7.8%) of ED patients reported compulsive stealing
Crisp et al. (49)	•Female patients diagnosed with AN (*n* = 102)	•Stealing occurred in 13.7% of patients; 4 patients were prosecuted •Those who acknowledged stealing were older, heavier at presentation, had never lost as much weight, and had been ill for longer than those who said they had never stolen •There was an almost exclusive positive association between stealing and the syndrome of bulimia/vomiting/purging
de la Serna de Pedro et al. (50)	•Patients diagnosed with BN (*n* = 45)	•11/45 (24.4%) of assessed BN patients presented with kleptomania symptomatology •6/19 (31.6%) of “primary” BN patients presented with kleptomania symptomatology •5/26 (19.2%) of “secondary” (mixed BN with restricting) BN patients presented with kleptomania symptomatology
Eddy et al. (51)	•Women seeking treatment for an eating disorder who also met DSM-III criteria for AN and/or BN (*n* = 246)	•13/136 (9.6%) of AN patients had a history of kleptomania •0/24 (0%) of AN-R “pure” (no lifetime history of binging or purging) participants had a history of kleptomania •2/27 (7%) of AN-R “not pure” (with a lifetime history of binging or purging at intake) participants had a history of kleptomania •11/85 (13%) of AN-BP participants had a history of kleptomania
Faber et al. (31)	•Study 1: obese adult women with (*n* = 84) and without (*n* = 113) BED •Study 2: compulsive buyers (*n* = 24) and “normal buying” age/sex-matched controls (*n* = 24)	•Study 1: women diagnosed with BED had significantly greater compulsive buying tendencies than non-binge eaters of similar weight •Study 2: compulsive buyers were more likely to have engaged in binge eating, have negative affect associated with weight and weight gain, exhibit more symptoms characteristic of BED and BN, and were more likely to be clinically diagnosed with an ED than matched controls
Fernández-Aranda et al. (52)	•Adult female patients with BN: without comorbid ICD (*n* = 173), with comorbid ICD (*n* = 54) •Pathological gamblers (*n* = 42)	•In BN, observed lifetime prevalence of ICD was 23.8% •Lifetime compulsive buying (17.6%) and intermittent explosive disorder (13.2%) were the most frequently reported ICDs •BN subtype was not significantly associated with lifetime ICD or ICD subtype •Those with BN and lifetime ICD presented more extreme personality profiles (especially on novelty seeking/impulsivity, general psychopathology) then those with BN without ICD
Fernández-Aranda et al. (12)	•Individuals aged 13–65 with BN purging type (proband) •Biological family members of proband, aged 13–65 with BN, AN, or EDNOS	•Lifetime ICD were present in 16.6%; compulsive buying disorder and kleptomania were the most common syndromes •ICD occurred more in individuals with binge eating subtypes, and was associated with significantly greater body image disturbance, higher harm avoidance, neuroticism, cognitive impulsivity, lower self-directedness, and greater use of laxatives, diuretics, appetite suppressants, and fasting •Those with ICD were more likely to have OCD, any anxiety disorder, specific phobia, depression, cluster B personality disorder, avoidant personality disorder, and to use psychoactive substances •Among those with ICD, 62% reported the ICD predated the ED and 45% reported the onset of both disorders within the same 3-year window
Fernández-Aranda et al. (36)	•Patients with EDs (*n* = 176) or gambling disorder (*n* = 184) receiving outpatient psychiatric treatment •Healthy controls (*n* = 151)	•Higher pathological buying prevalence was found in ED patients (12.5%) than those with gambling disorder (2.7%) and healthy controls (1.3%) •Female sex, higher impulsivity, and higher psychopathology were associated with pathological buying
Gerlinghoff and Backmund (53)	•Patients with an eating disorder (*n* = 63)	•Kleptomaniac behavior was found in 46/63 (73%) of all assessed ED patients •Kleptomaniac behavior was found in all patients with ANBN (37/37, 100%), and BN (4/4, 100%), and 5/22 (22.7%) of patients with AN •When kleptomaniac behavior is limited to occurring only in shops or from strangers then the following distribution occurs: 2/22 (9.1%) of AN patients, 10/37 (27.0%) of ANBN patients, and 3/4 (75%) of BN patients
Goldner et al. (54)	•ED group—women diagnosed with AN, BN, or EDNOS (*n* = 48) •Psychiatric control group—women accessing inpatient/outpatient psychiatry services (*n* = 46) •Control group—undergraduate women (*n* = 82)	•The 3 groups did not differ in overall history of shoplifting, but the ED women were more likely to have shoplifted in the past 6 months and to have shoplifted often than were women in the other two groups •Across all groups, current shoplifting was associated with low self-esteem, elevated depression, and purging behaviors at the time of assessment
Herzog et al. (55)	•Female patients seeking treatment for AN, BN, or mixed AN and BN (*n* = 229)	•7/229 (3%) of all ED patients assessed had a comorbid diagnosis of kleptomania •1/41 (2%) of AN patients had a comorbid diagnosis of kleptomania •1/98 (1%) of BN patients had a comorbid diagnosis of kleptomania •5/90 (5%) of mixed AN BN patients had a comorbid diagnosis of kleptomania
Hudson et al. (56)	•Individuals diagnosed with AN and/or BN at any point in their lifetime (*n* = 90) •First-degree relatives of 17 probands with schizophrenia (*n* = 41) •First-degree relatives of 15 probands with bipolar disorder (*n* = 50)	•Lifetime prevalence of ICD in the ED sample was 27/90 (30%) •25/90 (28%) of all ED patients had a lifetime diagnosis of kleptomania •2/16 (13%) AN patients, 11/25 (44%) ANBN patients, and 12/49 (24%) of BN patients had a lifetime diagnosis of kleptomania •2/90 (2%) of all ED patients had a lifetime diagnosis of intermittent explosive disorder •1/25 (4%) of ANBN patients and 1/49 (2%) of BN patients had a diagnosis of intermittent explosive disorder
Jiménez-Murica et al. (57)	•ED inpatients with (*n* = 25) and without (*n* = 1,656) pathological gambling (PG)	•Lifetime prevalence of PG was 1.49% •PG was associated with ED subtype; it was most common in BED (5.7%) and least in AN (0.6%) •ED+PG was more prevalent in males; these patients showed more impulsive behaviors, lower impulse regulation, and higher novelty seeking •Novelty seeking, being male, and higher BMI best predicted lifetime PG
Jiménez-Murica et al. (33)	•Female patients with: BN without comorbid compulsive buying (CB) (*n* = 50), BN with CB (*n* = 49), gambling disorder (*n* = 53), or CB (*n* = 36) •Non-psychiatric female controls (*n* = 50)	•Comorbid BN with CB was associated with highest eating psychopathology and social anxiety; they also displayed more dysfunctional personality traits and higher general psychopathology •On novelty seeking, the CB, gambling disorder, and BN with CB groups were similar to each other, whereas BN without CB presented a distinct profile •The clinical groups demonstrated higher overall levels of psychopathology compared to the control group •In comparison to the BN without CB group, BN with CB presented more severe eating psychopathology symptoms, as well as higher impulsivity and social impairment
		•Disorders with impulsive traits (CB, gambling disorder, BN with CB, and BN without BC) follow a linear trend in general psychopathology and specific personality traits, but differ along specific personality and psychopathological dimensions
Lacey and Read (58)	•Normal weight BN patients admitted to an inpatient program for multi-impulsive bulimia (*n* = 10)	•3/10 (33.3%) of BN patients reported stealing before treatment
Matsunaga et al. (27)	•Female patients with BN (*n* = 64)	•19/64 (30%) of BN subjects had a life-time incidence of shoplifting
Miyawaki et al. (59)	•Female patients with AN or BN seeking treatment for their eating disorder (*n* = 284)	•Lifetime prevalence of shoplifting among all assessed ED patients was 81/284 (28.5%) •12/99 (12.1%) AN-R patients reported lifetime shoplifting •19/72 (26.3%) of AN-BP patients reported lifetime shoplifting •50/113 (44.2%) of BN patients reported lifetime shoplifting
Nagata et al. (60)	•Consecutive outpatients treated between December 1996 and 1998 at the outpatient clinics of the Department of Neuropsychiatry, Osaka City University Hospital (*n* = 236) •Female nursing student control (*n* = 66)	•85/236 (36%) of all assessed ED participants reported repeated shoplifting (including food only) •3/60 (5%) AN-R participants reported shoplifting food only •16/62 (26%) AN-BP participants reported shoplifting food only •31/114 (27%) BN participants reported shoplifting food only •1/60 (2%) AN-R participants reported shoplifting (food excluded) •7/62 (11%) AN-BP participants reported shoplifting (food excluded) •27/114 (24%) BN participants reported shoplifting (food excluded)
Nagata et al. (61)	•Female AN and BN outpatients at the Department of Neuropsychiatry, Osaka City University Hospital (*n* = 185)	•38/185 (21%) of ED patients reported shoplifting •10/16 (63%) ED+DUD patients reported shoplifting •28/169 (17%) ED-DUD patients reported shoplifting
Nagata et al. (62)	•Patients with eating disorders and drug use disorders (*n* = 19) •Patients with methamphetamine use disorder (*n* = 12)	•39/183 (21%) of assessed ED participants reported repeated shoplifting •13/19 (68%) of ED + DUD patients reported repeated shoplifting •26/164 (16%) of ED only group reported repeated shoplifting
Nozoe et al. (63)	•Inpatients who completed treatment diagnosed with AN (*n* = 55)	•14/55 (25%) of all assessed AN participants reported a history of stealing after onset •0/9 (0%) of AN patients in group A (short stay at inpatient treatment) reported a history of stealing after onset •9/36 (25%) of AN patients in group B (medium stay at inpatient treatment) reported a history of stealing after onset •5/10 (50%) of AN patients in group C (long stay at inpatient treatment) reported a history of stealing after onset
Pryor et al. (64)	•Females presenting for evaluation at author's Eating Disorders Clinic between 1985 and 1994 (*n* = 171)	•22/171 (12.9%) of all AN participants reported having stolen food or weight related items •4/100 (4%) AN-R participants reported having stolen food or weight related items •18/71 (25%) AN-BP participants reported having stolen food or weight related items
Rowston and Lacey (65)	•Female normal-weight bulimics (*n* = 312)	•42% reported stealing on at least one occasion; this tended to occur later in the illness when binge-eating was fully established •Bulimic patients who stole had poor early interpersonal relationships, earlier onset sexual feelings, greater sexual activity, higher illicit drug use, and more obsessional qualities with increased ritualization
Takei et al. (66)	•Patients suffering with AN for 10 years of more (*n* = 16)	•8/13 (62%) AN patients assessed had kleptomania
Tanaka et al. (67)	•Patients admitted to receive inpatient treatment at Osaka City University Hospital between January 1982 and December 1999 with AN (*n* = 61)	•20/61 (32.8%) of AN patients reported shoplifting at referral
Vandereycken and Houdenhove (29)	•Females diagnosed with an ED (*n* = 155)	•73/155 (47.1%) of ED patients admitted to stealing
Weiss and Ebert (68)	•Normal weight females with BN (*n* = 15) •Normal weight female controls (*n* = 15)	•10/15 (67%) BN participants reported stealing •10/15 (67%) BN participants reported impulse buying
Wiederman and Pryor (30)	•Adult women diagnosed with BN-P (*n* = 217)	•86/217 (39.6%) BN patients reported stealing
Yip et al. (69)	•Patients with BED (*n* = 94)	•1.1% (*n* = 1) of patients met criteria for pathological gambling while 18.7% showed problem gambling features (at least 1 criteria for DSM-IV pathological gambling) •Men were more likely to have problem gambling features than women •BED patients with problem gambling features had lower self-esteem, greater substance use, and higher BMI (when controlling for gender)
Zucker et al. (70)	•Individuals with AN and or/ BN (*n* = 1,453)	•69/1,453 (5%) of ED participants engaged in repetitive hair pulling

*AN, anorexia nervosa; BN, bulimia nervosa; EDNOS, eating disorder not otherwise specified; EDE, Eating Disorder Examination; AN-R, anorexia nervosa restrictive subtype; OCD, obsessive-compulsive disorder; OCP, obsessive-compulsive personality; TAU, treatment as usual; BED, binge eating disorder; ICD, impulse control disorder; CBT-AN, cognitive behavior therapy for anorexia nervosa; CB, compulsive buying; DUD, Drug use disorder; ED + DUD, Eating disorder with comorbid drug use disorder; ED-DUD, Eating disorder without comorbid drug use disorder*.

### Participant Characteristics

A total of 9,646 individuals were identified as having an ED, ranging from sample sizes of 10 to 2,436 participants with EDs in individual studies. The mean age of individuals with EDs was 25.5 years (range 16.7–43.9), and the percentage of females was 97.7%.

### Risk-of-Bias

All studies included in this systematic review were evaluated with the modified Downs and Black instrument (Supplementary Table 1). For cross-sectional studies, the average Downs and Black score was 10.4/15, indicating predominantly moderate-quality studies. Although most studies were of moderate quality, several failed to account for the effects of important covariates such as age and gender.

### Prevalence of BAs and ICDs in Eating Disorders

**Figure 2:**
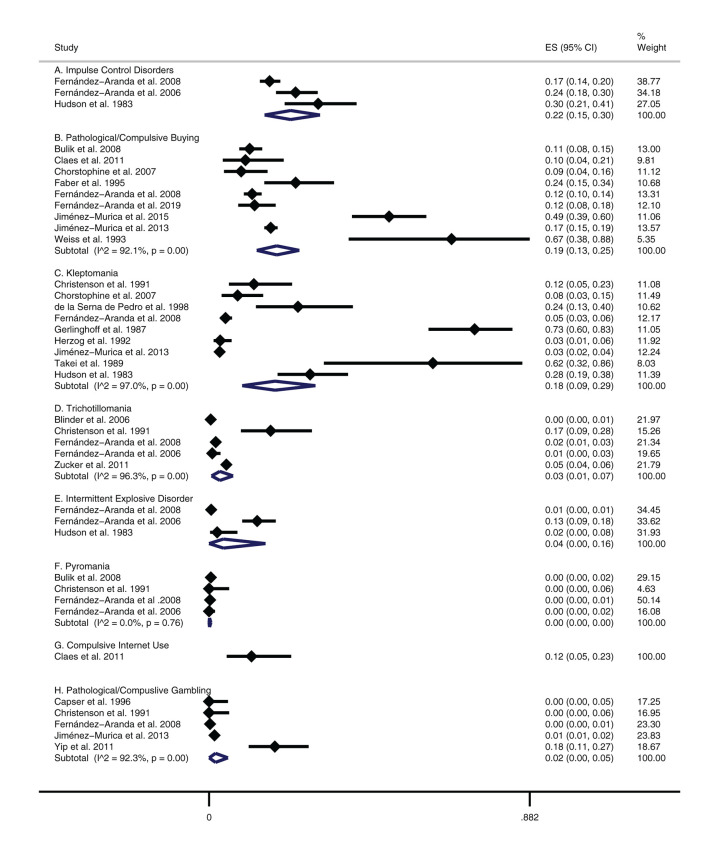
Prevalence of BAs/ICDs in patients with EDs.

In studies that looked at total ICDs/BAs, random pooled estimates demonstrated a 22% comorbid prevalence in those with EDs (3 studies, *N* = 1,026), see Figure 2. When examining individual types, pathological/compulsive buying had the highest prevalence at 19% in those with EDs (9 studies, *N* = 3,338), followed by a 18% prevalence for kleptomania (9 studies, *N* = 2,997), 4% prevalence for intermittent explosive disorder (3 studies, *N* = 1,026), 3% prevalence for trichotillomania (5 studies, *N* = 4,890), and a 2% prevalence for pathological/compulsive gambling (5 studies, *N* = 2,618). Pyromania did not appear to be prevalent in those with EDs (3 studies, *N* = 704).

In addition, there was a 12% prevalence for compulsive internet use, although this estimate was reported only on a single study and was imprecise (1 study, *N* = 60). Lastly, random pooled estimates demonstrated that stealing had a prevalence of 30% in those with EDs (13 studies, *N* = 3,749; see Supplementary Figure 1).

### Prevalence of BAs and ICDs by Eating Disorder Subtype

In studies that looked at total ICDs/BAs by ED subtype, there was a 24% prevalence in those with BN (1 study, *N* = 227), see Supplementary Figure 2. When examining individual ICDs/BAs by ED subtypes, compulsive buying had the highest prevalence at 42% in those with BN (3 studies, *N* = 341) followed by a 24% prevalence in those with BED (1 study, *N* = 84), an 11% prevalence in those with AN (1 study, *N* = 412). Kleptomania had the highest prevalence at 62% among those with AN (1 study, *N* = 13), followed by 11% among those with BN (3 studies, *N* = 337).

For individuals with BN, there was a 13% prevalence for intermittent explosive disorder (95% CI = 0.09–0.18; 1 study, *N* = 227), and a 3% prevalence for trichotillomania (2 studies, *N* = 292). For BED individuals, there was an 18% prevalence for pathological/compulsive gambling (1 study, *N* = 94). Neither pathological/compulsive gambling nor pyromania appeared to be prevalent in AN and BN studies.

The prevalence of stealing was 5% in AN-R (2 studies, *N* = 160), 19% for AN (4 studies, *N* = 630), 31% for AN-BP [anorexia nervosa binge eating/purging type (2 studies, *N* = 133), and 45% BN (4 studies, *N* = 658)], see Supplementary Figure 3.

## Discussion

### Summary

This systematic review and meta-analysis have contributed to the knowledge base by being the first review to examine the pooled prevalence of comorbid ICDs or BAs in individuals with EDs. Overall, it was estimated that nearly one-quarter (22%) of all people with EDs would experience an ICD at some point during their lives. Most ICDs were more prevalent among individuals with the binge/purge subtype of EDs, such as BN and AN-BP, relative to restrictive EDs, such as AN-R. In addition, roughly one-third (30%) of individuals with ED experienced stealing/shoplifting behaviors. The quality of the literature was rated as being moderate-quality across studies; however, several failed to report and adjust for potential confounders. Overall, we conclude that ICDs are common co-occurring conditions among individuals with EDs, and clinicians should be aware of the frequency of ICDs when providing care for people with EDs.

### Clinical Implications of Findings

The prevalence of comorbid ICDs/BAs, 22%, identified in this study closely resembles the prevalence of comorbid substance use disorders (SUDs) identified by a previous meta-analysis. The pooled prevalence was 22% (71). Clinicians must be aware of ICDs since they are associated with worse ED outcomes, particularly if they are undertreated (12, 52). In a similar vein, some personality traits (e.g., perfectionism, impulsivity, cognitive rigidity, and harm avoidance), psychiatric comorbidity, age of onset (e.g., early age of onset may contribute to poor prognosis), concurrent substance use disorders have been proposed as other risk factors that may play a role in deteriorating ED prognoses (72). Most notably, our meta-analysis identified an association between ICD and BN and AN-BP subtype compared to other ED diagnoses and ED behaviors. This supports previous literature indicating that individuals with binge-purge EDs (i.e., BN and AN-B) are more likely to struggle with ICDs, linked to more significant emotion regulation and impulse control difficulties (48, 73, 74).

Although our study did not identify and examine the differences in the comorbid ICDs/BAs ascertainment method, this may have contributed to statistical heterogeneity demonstrated between studies included in this review. For example, the DSM-III, DSM-IV, DSM-5, and the ICD have different specific criteria for other ICDs/Bas (6, 75–78). Furthermore, there are several self-report ED diagnostic instruments, such as the Eating Disorder Inventory (EDI) (79), and semi-structured interviews, such as the Eating Disorder Examination (EDE) (80). However, the reliability of these tools for detecting comorbid ICD/BA comorbidity may differ between studies using self-reported checklists (e.g., Eating Disorder Examination). In addition, individuals with ED often underestimate their impairments compared to informant reports (e.g., the information supplied by persons who are familiar with the patient, such as a supervisor, friend, partner, or family member), resulting in biased ICD/BA prevalence estimates (81, 82). Therefore, the diagnostic reliability could be improved further by incorporating collateral from multiple informants, especially when working with adolescents and young adults (83, 84). However, screening instruments will have a much better positive predictive value when the base rate, or prevalence of the condition, is higher in the population being screened. To that end, as this review provides an estimate of the prevalence of comorbid ICD/BAs, it can inform the best available base rate data for these disorders to date, which can support future epidemiological investigations.

Several studies have attempted to determine psychometrically robust tools for diagnosing comorbid ICDs, but there has generally been a poor correlation between rating scales (85–87). In addition, individuals may conceal the existence of ICDs/BAs due to shame and stigma, adding a layer of complexity to the screening process (88). For example, research has shown that individuals with gambling disorders experience stigma and are often stereotyped as selfish, greedy, or irresponsible, which may cause these individuals to delay help-seeking due to shame (89). Similarly, stigma research in individuals with AN has indicated that they believe the public trivializes their experiences by viewing their behaviors as within their control and by accrediting eating disorder behaviors solely to socio-cultural factors, which delays disclosing their illness (90). It may also be necessary to screen for ICDs and BAs in all EDs rather than EDs with bulimic-type psychopathology alone, as they appear to be comorbid with restrictive AN as well (e.g., pathological buying and kleptomania). As stratification by ED subtypes did not reconcile the heterogeneity in prevalence estimates, this suggests that the different instruments used to measure BAs/ICDs may differ (91–93).

One prospect for implementing and identifying treatment for individuals with these comorbid conditions is understanding the underlying pathophysiology of ICDs/BAs and the co-occurrence of SUDs. For example, both ICDs and BAs are postulated to share similar personality and neurobiological mechanisms (94). For instance, ICDs/BAs may share higher personality traits of impulsivity and sensation-seeking, with lower measures of harm avoidance (15). Furthermore, among women with co-occurring bulimia nervosa and ICDs, lower self-directedness, higher harm avoidance, and cooperativeness appear to be shared across disorders (12). In addition, research suggests that those with EDs and ICDs/BAs share a similar propensity for impulsivity (33, 95) and may have similar deficits in brain reward circuitry (34). Adding to this, it is worth noting that SUDs frequently co-occur with ICDs/BAS with the percentage of comorbid SUDs varying between 15 and 76% depending on the type of ICD/BA (96), and all conditions share similar characteristics of impulsivity. Thus, a transdiagnostic treatment approach targeting impulsivity in these comorbid conditions may help alleviate the burden that these individuals encounter, as there is mounting evidence supporting both pharmacological and behavioral treatments for impulsivity in SUDs (97), which has the potential to translate to efficacious therapies for individuals with EDs and comorbid ICDs/BAs. Finally, another potential option for treating ICDs/BAs in those with EDs is to take a personalized and flexible approach when treating those with EDs who have high levels of impulsivity (98).

### Strengths and Limitations

There are several strengths of this study. First, to our knowledge, this is the largest systematic review of ICDs among people with EDs and the first meta-analysis. Second, the quality of the majority of studies evaluated was fair. Third, the study was methodologically rigorous, comprehensive, and adhered to the MOOSE guidelines.

However, some limitations should be considered in the appraisal of the evidence presented by this review. One of the most considerable limitations is the limited number of studies available. Consequently, some prevalence estimates only involved one or two studies. Another significant limitation is the high heterogeneity observed across studies for most estimates. There are many potential sources to this heterogeneity, including the wide range of tools used to define ICDs and EDs, the range in study years included in the review, and different classifications of ICDs/BAs. However, a meta-regression was not performed to examine these potential sources of heterogeneity due to the limited number of studies included in the subgroup meta-analyses. While our study identified comorbid ICD diagnoses rather than symptoms, these estimates represent the presence of a clinically significant psychiatric comorbidity, which may be of more relevance to most clinicians. However, ICD/BA symptoms in the absence of a clinical diagnosis can still cause significant functional impairment, primarily if the individual endorses one symptom less than is required for a diagnosis. Furthermore, there may be differential associations between specific ICD/BAs symptoms and ED diagnoses/subtypes undetected in individual studies yet may be detected in a combined sample. As is demonstrated in prior studies and replicated in the current study, the ED symptoms of binge eating and/purging are essential in this association—and perhaps a similar finding exists at the ICD symptom level (74).

While sex proportions were reported in overall samples, sex-specific prevalence estimates were only reported by a subset of studies, precluding extensive sex-based analyses. Due to a gross underrepresentation of males in existing ED literature (99), few identified studies exploring ICD/BA comorbidity in males with ED. Hence, our study may have been underpowered to find sex differences. One meta-analysis of community studies reported the 12-month and lifetime prevalence of EDs as 2.6- and 4.2-fold higher among women than men (100). Conversely, most ICD/BA diagnoses (i.e., pyromania, gambling disorder, intermittent explosive disorder, and oppositional defiant disorder) occur more frequently in men, except for kleptomania, which occurs three times more frequently among women (101, 102). Accordingly, while we found no relevant prevalence estimates of gambling disorder in BN, an ED predominantly diagnosed in women, studies reported an 18% prevalence in BED, an ED characterized by a more balanced woman-to-men ratio. Studies examining gender differences in compulsive buying have yielded mixed results. In a German community sample (103), compulsive buying occurred equally between men and women. However, women in a Spanish community sample showed a higher propensity for compulsive buying (104). Additionally, while upwards of 80% of compulsive buyers seeking treatment are women, this may reflect the notion that women are more likely to recognize and seek help for the problem rather than true differences in prevalence between genders (105). These findings might explain the differences in prevalence for the different types of ICDs/BAs in the current study samples of primarily women (97.8%) with EDs. Another aspect that may explain the differences in prevalence might be that the incidence of ICDs/BAs also tends to vary with age. For example, while oppositional defiant disorder and conduct disorder have the most significant incidence before adolescence (101), intermittent explosive disorder occurs at any point under age 40 (106).

To that end, as this is a study-level meta-analysis, a limitation of the methods is that individual-level characteristics were not explored. There was also a limited representation of studies from all geographic regions, which limited our ability to estimate the prevalence of comorbid ICD/BAs across all continents. There was little information about ICDs/BAs in specific EDs, such as night-eating syndrome, ARFID, or atypical anorexia. Consequently, the overall prevalence estimates presented in the current study are not representative of all individuals with ED, but rather AN, BN, and BED primarily. Finally, as with all meta-analyses, we were limited by the quality and quantity of existent studies, and our results reflect only what is available in terms of existing literature.

### Future Research

Future research is needed to understand better the prevalence of ICD/BAs in subgroups of ED individuals, especially males, children and adolescents, and older adults. Adolescents and young adults are especially important groups regarding ICD/BAs as this age group overlaps with a neurobiologically sensitive period for developing impulse control. This characteristic appears to influence the course of these disorders. Finally, given the bidirectional relationship between ICDs/BAs and EDs, and related conditions (55, 74, 107–110)—such as substance use disorders (SUDs) and personality disorders (PD), future research should examine the corresponding prevalence of these comorbidities in alternative primary populations (e.g., the prevalence of EDs in people with ICDs/BAs). In addition, future studies could examine the bidirectional causation by conducting longitudinal studies that examine EDs and ICDs/BAs. Future research directions might also involve additional studies exploring treatment options for individuals with co-occurring EDs and ICDs/BAs, particularly pharmacological interventions, rTMS, or combining the two with or without concomitant psychotherapies.

## Conclusions

This is the first meta-analysis on the comorbid prevalence of EDs and ICDs/BAs. We found a moderate prevalence for these comorbid conditions, with approximately one out of five individuals with an ED also displaying a comorbid ICD/BA, with differences among subtypes. Although causal inferences cannot be drawn, the numbers strongly suggest that clinical screening/monitoring of ICDs/BAs should be part of the clinical routine in cohorts with EDs. ED settings need either the capacity to manage these disorders or adequate access to relevant services. Further investigations are needed to reveal common underlying pathomechanisms.

## Data Availability Statement

The raw data supporting the conclusions of this article will be made available by the lead author upon reasonable request.

## Author Contributions

DD, GD, SP, and GP contributed to conception and design of the study. DD organized the methods and conducted the meta-analysis and wrote the first draft of the manuscript. MS, AB, and GP wrote sections of the manuscript. AA, AS, AR, and JF screened titles, abstracts, and papers. AA, AS, AR, JF, and CR extracted data for tables, figures, and the meta-analysis. All authors contributed to manuscript revision, read, and approved the submitted version.

## Funding

This work was supported by the O'Brien Institute for Public Health and Mathison Center for Mental Health Post-doctoral Scholarship, University of Calgary Cumming School of Medicine Post-doctoral Scholarship, and the Harley Hotchkiss - Samuel Weiss Postdoctoral Fellowship awarded to DD, the Cuthbertson and Fischer Chair in Pediatric Mental Health awarded to SP, and the Alberta Children's Hospital Foundation and the Alberta Children's Hospital Research Institute via GD.

## Conflict of Interest

The authors declare that the research was conducted in the absence of any commercial or financial relationships that could be construed as a potential conflict of interest.

## Publisher's Note

All claims expressed in this article are solely those of the authors and do not necessarily represent those of their affiliated organizations, or those of the publisher, the editors and the reviewers. Any product that may be evaluated in this article, or claim that may be made by its manufacturer, is not guaranteed or endorsed by the publisher.
