# Supplementary material for: The Prevalence of Impulse Control Disorders and Behavioral Addictions in Eating Disorders: A Systematic Review and Meta-Analysis
Source: Front Psychiatry. 2022 Jan 6;12:724034. doi: 10.3389/fpsyt.2021.724034 (PMC8770943; doi:10.3389/fpsyt.2021.724034)
Supplement: Supplementary file 1 [file Data_Sheet_1.doc]

**Supplementary Material:** Searches

Database: Ovid MEDLINE(R) and Epub Ahead of Print, In-Process, In-Data-Review & Other Non-Indexed Citations and Daily <1946 to May 06, 2021>

Search Strategy:

--------------------------------------------------------------------------------

1. exp "Feeding and Eating Disorders"/ (31814)
2. (anorex* nervosa or bulim* nervosa or binge* eat* or purg* or ((eating or feeding or food or appetite or purging) adj2 disorder*) or Avoidant Restrictive Food Intake Disorder or OSFED or ARFID).tw,kf. (42515)
3. 1 or 2 (51190)
4. exp impulse control/ or kleptomania/ or pyromania/ or intermittent explosive disorder/ or pathological gambling/ or trichotillomania/ or compulsive buying/ or compulsive sexual behavior/ (9185)
5. ((compulsive or pathologic*) adj2 (buying or shopping or purchasing or sexual* or internet)).tw,kf. (1008)
6. (impuls* control or impulse control disorder* or kleptomani* or pyromani* or intermittent explosive disorder* or pathologic* gambling or trichotillomani*).tw,kf. (6971)
7. 4 or 5 or 6 (13515)
8. 3 and 7 (592)

***************************

Database: APA PsycInfo <1806 to May Week 1 2021>

Search Strategy:

--------------------------------------------------------------------------------

1. exp eating disorders/ or anorexia nervosa/ or binge eating disorder/ or bulimia/ or feeding disorders/ or hyperphagia/ or kleine levin syndrome/ or pica/ or "purging (eating disorders)"/ or "rumination (eating)"/ or aphagia/ or binge eating/ or food addiction/ (33459)
2. (anorex* nervosa or bulim* nervosa or binge* eat* or purg* or ((eating or feeding or food or appetite or purging) adj2 disorder*) or Avoidant Restrictive Food Intake Disorder or OSFED or ARFID).ti,ab,id. (37679)
3. 1 or 2 (41681)
4. exp impulse control disorders/ or behavioral disinhibition/ or impulsiveness/ or kleptomania/ or pyromania/ or intermittent explosive disorder/ or pathological gambling/ or trichotillomania/ or compulsive buying/ or compulsive sexual behavior/ (16543)
5. ((compulsive or pathologic*) adj2 (buying or shopping or purchasing or sexual* or internet)).ti,ab,id. (1628)
6. (impuls* control or impulse control disorder* or kleptomani* or pyromani* or intermittent explosive disorder* or pathologic* gambling or trichotillomani*).ti,ab,id. (8403)
7. 4 or 5 or 6 (21214)
8. 3 and 7 (940)

***************************

Database: Embase <1974 to 2021 May 06>

Search Strategy:

--------------------------------------------------------------------------------

1. exp eating disorder/ or anorexia nervosa/ or avoidant restrictive food intake disorder/ or binge eating disorder/ or bulimia/ or emotional eating/ or food addiction/ or food aversion/ or food refusal/ or orthorexia/ or pica/ or purging disorder/ (54946)
2. (anorex* nervosa or bulim* nervosa or binge* eat* or purg* or ((eating or feeding or food or appetite or purging) adj2 disorder*) or Avoidant Restrictive Food Intake Disorder or OSFED or ARFID).tw,kw. (54730)
3. 1 or 2 (71021)
4. exp impulse control disorder/ or "autophagia (mental disorder)"/ or intermittent explosive disorder/ or kleptomania/ or pathological gambling/ or pyromania/ or trichotillomania/ or compulsive buying/ or compulsive sexual behavior/ or behavioral disinhibition/ (12565)
5. ((compulsive or pathologic*) adj2 (buying or shopping or purchasing or sexual* or internet)).tw,kw. (1657)
6. (impuls* control or impulse control disorder* or kleptomani* or pyromani* or intermittent explosive disorder* or pathologic* gambling or trichotillomani*).tw,kw. (10291)
7. 4 or 5 or 6 (17332)
8. 3 and 7 (1427)

***************************

Database: CINAHL

Search Strategy:

--------------------------------------------------------------------------------

1. (MH "Eating Disorders+") OR (MH "Feeding and Eating Disorders of Childhood+") OR (MH "Binge Eating Disorder") OR (MH "Bulimia Nervosa") OR (MH “Anorexia Nervosa”) OR (MH “Purging Disorder”) (19,102)
2. TI ( anorex* nervosa OR bulim* nervosa OR binge* eat* OR purg* OR ARFID OR OSFED OR ((eating or feeding or food or appetite) N2 disorder*) ) OR AB ( anorex* nervosa OR bulim* nervosa OR binge* eat* OR purg* OR ARFID OR OSFED OR ((eating or feeding or food or appetite) N2 disorder*) ) OR SU ( anorex* nervosa OR bulim* nervosa OR binge* eat* OR purg* OR ARFID OR OSFED OR ((eating or feeding or food or appetite) N2 disorder*) ) (21,469)
3. S1 OR S2 (23,840)
4. (MH "Impulse Control Disorders+") OR (MH “Autophagia”) OR (MH “Intermittent Explosive Disorder”) OR (MH “Kleptomania”) OR (MH “Pathological Gambling”) OR (MH “Pyromania”) OR (MH “Trichotillomania”) OR (MH “Compulsive Buying”) OR (MH “Compulsive Sexual Behavior”) (4,749)
5. TI ( impuls* control OR Impulse control disorder* OR kleptomani* OR pyromani* OR intermittent explosive disorder* OR pathological gambling OR trichotillomani* OR (compulsive N2 (buying or shopping or purchasing or sexual*)) ) OR AB ( impuls* control OR Impulse control disorder* OR kleptomani* OR pyromani* OR intermittent explosive disorder* OR pathological gambling OR trichotillomani* OR (compulsive N2 (buying or shopping or purchasing or sexual*)) ) OR SU ( impuls* control OR Impulse control disorder* OR kleptomani* OR pyromani* OR intermittent explosive disorder* OR pathological gambling OR trichotillomani* OR (compulsive N2 (buying or shopping or purchasing or sexual*)) ) (5,660)
6. S4 Or S5 (8,165)
7. S3 AND S6 (351)

***************************

**Supplementary Table 1**. Downs & Black Risk of Bias Assessment for Cross-Sectional Studies

| Study (First Author, Year) | 1.Aims | 2.Measures | 3.Characteristics | 5.Confounders | 6.Findings | 7.Random variability | 10.Probabilities | 11.Representative sample | 12.Representative accepted | 13.Standard facilities | 16.Data dredging | 18.Statistics | 20.Accurate measures | 25.Confond adjustment | **Total** |
| --- | --- | --- | --- | --- | --- | --- | --- | --- | --- | --- | --- | --- | --- | --- | --- |
| Blinder 2006 | 1 | 1 | 1 | 2 | 1 | 1 | 1 | U | U | 1 | 1 | 1 | 1 | 1 | **13** |
| Bulik 1991 | 1 | 1 | 1 | 2 | 1 | 1 | 1 | 1 | U | 1 | 1 | 1 | 1 | 1 | **14** |
| Christenson, 1990 | 1 | 1 | 1 | 0 | 1 | 1 | 1 | U | 0 | 1 | 1 | 1 | 1 | 0 | **10** |
| Claes, 2011 | 1 | 1 | 1 | 0 | 1 | 1 | 0 | U | 0 | U | 1 | 1 | 1 | 0 | **8** |
| Corstorphine 2004 | 1 | 1 | 1 | 0 | 1 | 1 | 1 | 1 | 1 | 0 | 1 | 1 | 1 | 0 | **11** |
| Crisp, 1980 | 1 | 0 | 1 | 0 | 1 | 1 | 0 | U | U | 1 | 0 | 1 | 1 | 0 | **7** |
| De la Serma de Pedro 1998 | ? | ? | ? | ? | ? | ? | ? | ? | ? | ? | ? | ? | ? | ? | **?** |
| Faber, 1995 | 1 | 1 | 1 | 1 | 1 | 0 | 0 | 1 | U | 1 | 1 | 1 | 1 | 1 | **11** |
| Fernandez-Aranda, 2006 | 1 | 1 | 1 | 1 | 1 | 1 | 1 | 1 | 0 | 1 | 1 | 1 | 1 | 1 | **13** |
| Fernandez-Aranda, 2008 | 1 | 1 | 1 | 0 | 1 | 1 | 1 | 1 | U | 1 | 1 | 1 | 1 | 0 | **11** |
| Fernandez-Aranda, 2019 | 1 | 1 | 1 | 1 | 1 | 1 | 1 | 1 | 1 | 1 | 1 | 1 | 1 | 1 | **14** |
| Gerlinghoff 1987 | 1 | 1 | 1 | 0 | 1 | 0 | 0 | U | U | 0 | 1 | 1 | 1 | 0 | **7** |
| Goldner, 2000 | 1 | 1 | 1 | 0 | 1 | 1 | 0 | U | 0 | 1 | 1 | 1 | 1 | 0 | **9** |
| Hudson 1983 | 1 | 1 | 1 | 0 | 1 | 0 | 0 | 1 | 1 | 1 | 1 | 1 | 1 | 0 | **10** |
| Jimenez-Murcia, 2013 | 1 | 1 | 1 | 0 | 1 | 1 | 1 | 1 | 1 | 1 | 1 | 1 | 1 | 0 | **12** |
| Jimenez-Murcia, 2014 | 1 | 1 | 1 | 1 | 1 | 1 | 1 | 1 | 1 | 1 | 1 | 1 | 1 | 1 | **14** |
| Matsunaga 1998 | 1 | 1 | 1 | 0 | 1 | 1 | 1 | U | U | 1 | 1 | 1 | 1 | 0 | **10** |
| Miyawaki 2018 | 1 | 1 | 1 | 1 | 1 | 1 | 1 | 1 | 1 | 1 | 1 | 1 | 1 | 0 | **13** |
| Nagata 2002 | 1 | 1 | 1 | 2 | 1 | 1 | 1 | 1 | U | 1 | 1 | 1 | 1 | 1 | **14** |
| Nagata et al. 2000 | 1 | 1 | 1 | 0 | 1 | 1 | 1 | U | U | U | 1 | 1 | 1 | 0 | **9** |
| Nagata et al. 2003 | 1 | 1 | 1 | 0 | 1 | 1 | 1 | U | U | U | 1 | 1 | 1 | 0 | **9** |
| Nozoe 1995 | 1 | 1 | 1 | 0 | 1 | 0 | 0 | 1 | 1 | 1 | 1 | 1 | 1 | 0 | **10** |
| Pryor 1995 | 1 | 1 | 1 | 0 | 1 | 1 | 1 | U | U | 0 | 1 | 1 | 1 | 0 | **9** |
| Rowston, 1992 | 1 | 1 | 0 | 0 | 1 | 0 | 1 | U | U | 1 | 1 | 1 | 1 | 0 | **8** |
| Takei 1989 | 1 | 1 | 1 | 0 | 1 | 1 | 0 | 0 | 0 | U | 1 | 1 | 1 | 0 | **8** |
| Zucker 2011 | 1 | 1 | 1 | 1 | 1 | 1 | 1 | U | U | U | 1 | 1 | 1 | 1 | **11** |
| Vandereycken 1996 | 1 | 1 | 1 | 0 | 1 | 1 | 1 | U | U | 0 | 1 | 1 | 1 | 0 | **9** |
| Weiss 1983 | 1 | 1 | 1 | 0 | 1 | 1 | 0 | 0 | 0 | 0 | 1 | 1 | 1 | 0 | **8** |
| Wiederman 1995 | 1 | 1 | 0 | 0 | 1 | 1 | 1 | U | U | 0 | 1 | 1 | 1 | 0 | **8** |
| Casper 1996 | 1 | 1 | 1 | 1 | 1 | 1 | 0 | 1 | U | 1 | 1 | 1 | 1 | 0 | **11** |
| Eddy et al. 2002 | 1 | 1 | 1 | 1 | 1 | 0 | 1 | 1 | 1 | 1 | 1 | 1 | 1 | 0 | **12** |
| Herzog 1991 | 1 | 1 | 1 | 1 | 1 | 1 | 1 | 1 | U | 1 | 1 | 1 | 1 | 1 | **13** |
| Tanaka 2001 | 1 | 1 | 1 | 0 | 1 | 1 | 1 | 1 | 1 | 1 | 1 | 1 | 1 | 0 | **12** |
| Lacey 1993 | 1 | 0 | 1 | 0 | 1 | 0 | 0 | U | U | U | 1 | 1 | 1 | 0 | **6** |
| Yip, 2011 | 1 | 1 | 1 | 0 | 1 | 1 | 1 | U | U | 1 | 1 | 1 | 1 | 0 | **10** |
| **Total** | **34** | **32** | **32** | **15** | **34** | **27** | **23** | **16** | **9** | **22** | **33** | **34** | **34** | **9** |  |

**Supplementary Figure 1:** Prevalence of Stealing/Shoplifting Across All EDs

**Supplementary Figure 2:** Prevalence of ICDs/BAs by ED Subtype


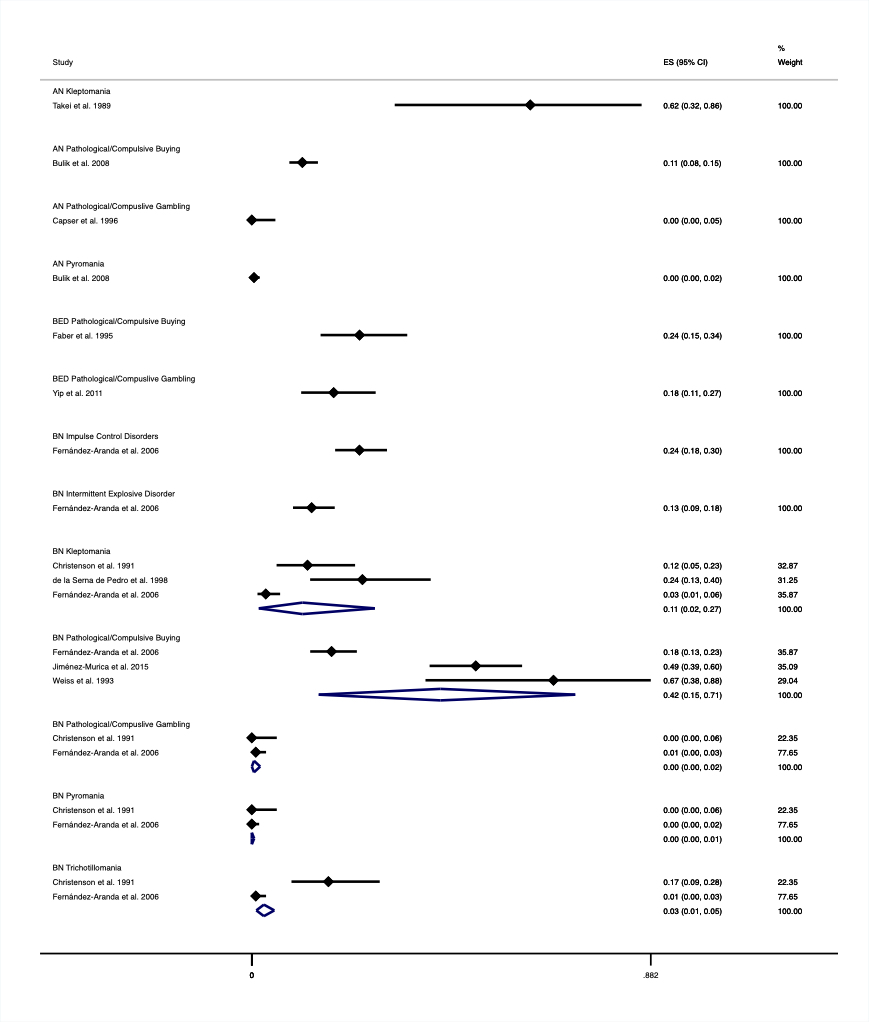


**Supplementary Figure 3:** Prevalence of Stealing/Shoplifting by ED Subtype
